# Weighing up the evidence on ethanol excipient use in common neonatal medications

**DOI:** 10.1038/s41390-025-04211-w

**Published:** 2025-06-17

**Authors:** Kelly Q. Zhou, Eleanor R. Gunn, Laura Millward, Joanne O. Davidson, Justin M. Dean, Laura Bennet, Alistair J. Gunn

**Affiliations:** 1 Department of Physiology, The University of Auckland, Auckland, New Zealand; 2 Neonatal intensive care, Middlemore Hospital, Auckland, New Zealand; 3 Women’s and Children’s Pharmacy, Middlemore Hospital, Auckland, New Zealand

Moderate to heavy maternal prenatal alcohol consumption is highly associated with adverse developmental outcomes in their offspring.^1,2^ There is still conflicting evidence on the effects of “light” drinking during pregnancy on behavioral or cognitive development in mid-childhood.^3^ There is currently no safe recommended level of alcohol exposure during pregnancy.^4,5^ In a systematic review of fetal alcohol spectrum disorder, clinical features of fetal alcohol spectrum disorder including reduced head circumference, low birth weight, and sentinel facial features were all highly correlated with increasing prenatal alcohol exposure. However, evidence for the effects of low prenatal alcohol exposure were inconsistent.^2^ The global rate of fetal alcohol spectrum disorder is 0.77% with significant variation by country and community.^6^ Research into fetal alcohol spectrum disorder is challenging due to the need to rely on cohort and case-controlled studies with risk of confounding and recall bias. However, it is highly likely that both the timing of prenatal alcohol exposure, the dose and cumulative exposure are all associated with clinical impact.^7,8^

In preclinical studies in the developing brain, ethanol exposure has had mixed effects. In preterm and term equivalent fetal sheep, intoxicating levels of maternal ethanol are associated with multiple deleterious effects including severe white matter injury.^9,10^ However, in preterm fetal sheep, maternal administration of 2% ethanol (~ 0.7 g) before umbilical cord occlusion was associated with improved neuronal survival in the caudate nucleus, but greater neuronal loss in some regions of the hippocampus and reduced proliferation and increased microglia in the white matter.^11^ Strikingly, in neonatal piglets, the combination of hypothermia (1–13 h) and 2.5% ethanol (7.2 ml/kg/h) at 1–3 h and 25–27 h after carotid artery occlusion and hypoxia, was associated with greater oligodendrocyte survival than with hypothermia alone.^12^ These mixed effects in the setting of hypoxic-ischemic brain injury strongly suggest that the impact of ethanol is likely to be maturation- and situation- specific.

The United States Food and Drug Administration (FDA) approved amount of ethanol as an excipient in oral over-the-counter products is 0.5% for children under 6 years of age. The American Academy of Pediatrics Committee on Drugs suggests that blood ethanol concentration should not exceed 25 mg/100 mL following a single dose of medication, whereas the European Medicines Agency (EMA) approved amount is based on blood ethanol levels not exceeding 1 mg/dL after a single dose, or a dose not exceeding 6 mg/kg in children under 6 years of age.^13^ By contrast, the threshold for safe ethanol exposure for preterm newborns, if any, is unknown.^14^

Preterm infants in the neonatal intensive care unit (NICU) are commonly exposed to drugs that use ethanol as an excipient either as a solvent or a preservative. Mahajan et al., in this issue, collected urine samples from preterm infants who had received ferrous sulfate and/or chlorothiazide formulations that contain ethanol as an excipient.^15^ This study measured the ethanol biomarkers ethyl sulfate (EtS) and ethyl glucuronide (EtG) in the urine, as blood ethanol levels could not be measured directly. They showed greater accumulation of ethanol urine metabolites with decreasing gestational age, raising concern for the toxicity of ethanol in this vulnerable group. However, the clinical effects of this level of ethanol exposure are not known. Extremely preterm infants have increased rates of neurodevelopmental impairment compared to term-born infants, including greater rates of autism spectrum disorder, attention deficit hyperactivity disorder and expressive language delay – disorders that may also be associated with fetal alcohol spectrum disorder, making it more difficult to tease out the contribution of alcohol exposure to neurodevelopmental outcomes in preterm infants.^16,17^

Mahajan et al. report that the neonates in the study received between 0.04 – 3 mg/kg/day of ethanol for between 2 and 65 days.^15^ In early ethanol toxicity studies, juvenile rodents were often exposed to much higher doses, of at least 5 g/kg/day.^18,19^ 7.6 g/kg/day was associated with the development of microcephaly in neonatal rat pups. Interestingly, blood ethanol levels varied considerably among pups receiving the same dose of ethanol, and the extent of brain growth reduction was more associated with blood ethanol levels than the dose of ethanol.^19^ Better knowledge of actual blood ethanol levels in neonates who have received medications containing ethanol would help to guide preclinical studies to assess more subtle neurodevelopmental effects at equivalent blood ethanol levels in animals. If urinary ethanol metabolites can be shown to correlate with blood alcohol levels, this could be a valuable biomarker for monitoring levels of ethanol exposure in preterm infants. Sick preterm infants are at higher risk of acute kidney impairment and neonatal cholestasis – conditions that may affect clearance of ethanol and ethanol byproducts.^20,21^ Further, sick extremely preterm infants are more likely to receive multiple different medications containing alcohol excipients.^22^ Therefore, dose response, the effect of acute versus chronic exposure and the impact of acute kidney injury and hepatic dysfunction should be systemically investigated to establish safe ethanol exposure levels in these populations. This information is crucial, as the benefits of administration of a drug containing ethanol must be considered against the potential adverse effects of ethanol.

Several studies (European Study for Neonatal Excipient Exposure and the Safe Excipient Exposure in Neonates and Small Children study) have highlighted that newborns are often exposed to ethanol in doses over the recommended adult dosing, as well as other potentially toxic excipients such as propylene glycol, parabens, polysorbate 80, benzoates, saccharin sodium, sorbitol, and benzalkonium chloride.^23,24^ These studies have provided guidance on how to limit or mitigate exposure to these excipients. It is vital to clearly state the concentrations of potentially toxic excipients including ethanol. The regulations for disclosing the concentration of excipients may differ between countries. In some medications it is difficult to find the exact concentration of ethanol. For example, in ferrous sulfate (Ferodan), the ethanol is contained in a flavoring agent (<0.15% w/v), so we can assume that the overall ethanol content is <0.15% w/v. The regulations for disclosing the concentration of excipients may differ between countries. Additionally, for the same active compound, there can be different formulations which contain different excipients or different concentrations of the same or different excipient. This information should be readily available to enable clinicians to make an informed decision on which formulation to best prescribe.

Mahajan et al., have highlighted that preterm infants of decreasing gestational age have greater accumulation of ethanol from medications containing ethanol as an excipient.^15^ If urinary ethanol byproducts are shown to correlate with blood ethanol levels, these biomarkers could provide a valuable non-invasive method to monitor ethanol exposure in these vulnerable preterm infants, guiding reductions in ethanol excipient exposures, and potentially helping to tease out the differential effects of ethanol exposure and prematurity on long-term neurodevelopmental outcomes. In the absence of clear information on the impact of relevant concentrations, on a precautionary basis we should try to minimize exposure of ethanol in preterm infants.
